# Macrophages and Immune Responses in Uterine Fibroids

**DOI:** 10.3390/cells10050982

**Published:** 2021-04-22

**Authors:** Alessandro Zannotti, Stefania Greco, Pamela Pellegrino, Federica Giantomassi, Giovanni Delli Carpini, Gaia Goteri, Andrea Ciavattini, Pasquapina Ciarmela

**Affiliations:** 1 Department of Specialist and Odontostomatological Clinical Sciences, Università Politecnica delle Marche, 60126 Ancona, Italy; a.zannotti@pm.univpm.it (A.Z.); g.dellicarpini@staff.univpm.it (G.D.C.); a.ciavattini@univpm.it (A.C.); 2 Department of Experimental and Clinical Medicine, Università Politecnica delle Marche, 60126 Ancona, Italy; s.greco@pm.univpm.it (S.G.); p.pellegrino@pm.univpm.it (P.P.); 3 Department of Biomedical Sciences and Public Health, Università Politecnica delle Marche, 60126 Ancona, Italy; f.giantomassi@virgilio.it (F.G.); g.goteri@staff.univpm.it (G.G.)

**Keywords:** uterine fibroids, ECM, inflammatory process, tissue repair, macrophages, pathological fibrosis

## Abstract

Uterine fibroids represent the most common benign tumors of the uterus. They are considered a typical fibrotic disorder. In fact, the extracellular matrix (ECM) proteins—above all, collagen 1A1, fibronectin and versican—are upregulated in this pathology. The uterine fibroids etiology has not yet been clarified, and this represents an important matter about their resolution. A model has been proposed according to which the formation of an altered ECM could be the result of an excessive wound healing, in turn driven by a dysregulated inflammation process. A lot of molecules act in the complex inflammatory response. Macrophages have a great flexibility since they can assume different phenotypes leading to the tissue repair process. The dysregulation of macrophage proliferation, accumulation and infiltration could lead to an uncontrolled tissue repair and to the consequent pathological fibrosis. In addition, molecules such as monocyte chemoattractant protein-1 (MCP-1), granulocyte macrophage-colony-stimulating factor (GM-CSF), transforming growth factor-beta (TGF-β), activin A and tumor necrosis factor-alfa (TNF-α) were demonstrated to play an important role in the macrophage action within the uncontrolled tissue repair that contributes to the pathological fibrosis that represents a typical feature of the uterine fibroids.

## 1. Uterine Leiomyoma: A Typical Fibrotic Pathology

Uterine leiomyomas (leiomyomas, myomas, uterine fibroids, fibroids) are the most common benign tumors of the uterus. The perimetrium constitutes the more external layer of the uterus; it equals the peritoneum and is surrounded by a thin connective tissue layer. The perimetrium resembles a typical serosa/adventitia layer. The endometrium constitutes the more internal layer. It is formed by a simple columnar epithelium and contains numerous tubular glands. In addition, a cell-dense connective tissue layer can be individuated at the level of this structure. Finally, a transition to squamous non-keratinized epithelium at the portio (squamocolumnar junction) can be appreciated. Functionally, the endometrium can be divided into two sublayers: the so-called stratum basale, which represents the basal layer, and the so-called stratum functionale, which is the real functional layer. The endometrium resembles a typical mucosa layer. Finally, the myometrium constitutes the intermediate layer between the perimetrium and the endometrium and represents the muscularis structure of the uterus. The uterine musculature shows properly the typical characteristics of the smooth muscle tissue. More precisely, the myometrium is composed of three smooth muscle layers: the subvascular layer, which is quite thin; the vascular layer, which is rather strong and well-perfused; and the supravascular layer, which is composed of a complex of crossing muscle fibers. The subvascular layer is mainly involved in the separation of the endometrium during the menstrual cycle. The vascular layer runs around the uterus and, in doing this, it forms a kind of net for the perfusion of the tissue. It plays a major role during labor within the complex mechanism that regulates the uterine contractions during the partum [1]. The supravascular, with its muscle fibers, stabilizes the uterine wall [2,3]. The cells of the myometrium can transform themselves into uterine leiomyoma cells. So, the uterine leiomyoma is a pathology that involves, in detail, the myometrium. The uterine fibroids incidence in reproductive age women is approximately 60%, and if we consider black women, this percentage reaches 80% [4]. The symptomatology of uterine fibroids is very heavy. One of the most relevant clinical symptoms is prolonged or heavy menstrual bleeding. In addition, the irregular and excessive bleeding often experienced by the women affected by uterine leiomyomas, a lot of times, leads to anemia. Other symptoms of the uterine fibroids are represented by pelvic pain or pressure, pain at the level of the back of the legs, a pressure sensation at the level of the lower part of abdomen, bowel and bladder dysfunctions and pain during sexual intercourse.

In addition to all these physical ailments, uterine leiomyomas may also impact the pregnancy outcome. Depending on their position, size and number, uterine leiomyomas can be a cause of infertility and recurrent miscarriage [5,6,7,8,9]. Although uterine leiomyomas are not malignant tumors, they can cause significant morbidity. Thus, this pathology represents one of the most important public health problems worldwide [10]. This fact becomes also more relevant if we bear in mind that, at the moment, no long-term medical treatments are available for fibroids resolution [11].

Considering the role played by estrogens and progesterone in the leiomyoma growth [11,12], for the treatment of uterine fibroids, the U.S. Food and Drug Administration (FDA) approved leuprolide acetate, which is a gonadotropin-releasing hormone analog. However, these kinds of molecules, in particular in young women, can provoke several side effects, above all, a hypogonadal state; this is the reason why the duration of therapy is currently limited. Uterine leiomyomas usually start to grow again after breaking off the treatment [13,14]. Nevertheless, it was demonstrated that leuprolide acetate can be effectively used in order to decrease the volume of the uterine fibroids with improved fibroid-related symptoms. [15,16,17]. Of the treatments that have been studied up to now, the focus has above all been on those belonging to two categories: antiprogestin and selective progesterone receptor modulators (SPRMs). Thus, clinical trial results suggested mifepristone, which is an antiprogestinic molecule [18], and asoprisnil [19] and telapristone acetate (CDB-4124) [20], both belonging to the SPRMs category, as candidate therapeutic drugs against uterine fibroids (https://clinicaltrials.gov accessed on 7 April 2021). In particular, 17a-acetoxy-11b-(4-*N*,*N*-dimethylaminophenyl)-19-norpregna-4,9-diene-3,20-dione, also referred to as CDB-2914 and ulipristal acetate (UPA) [21,22,23,24], is an SPRM molecule, and it is very interesting to study because of the high affinity that it has shown in binding progesterone receptor isoforms A and B [25,26].

Currently, in the international literature, there is a debate about the usefulness and safety of the use of UPA [27].

A few years ago, we demonstrated that UPA can exert a downregulation effect at the level of the mRNA of activin A, a pro-fibrotic factor for leiomyoma. The UPA causes a similar impairing effect also on follistatin (FST), activin receptor type II (ActRIIB) and activin receptor-like kinase 4 (ALK4) mRNAs [28]. All these molecules together represent the activin pathway and these results consider activin A and its receptors as UPA targets and at the same time reinforce the validity of UPA as a treatment for uterine fibroids.

In 2012, the European Medicines Agency (EMA) approved the clinical use of UPA 5 mg, sold under the trade name Esmya (or generic medicines), but limited it to a three-month period and pre-surgery. However, in 2018, other limitations occurred since cases of severe liver toxicity had been reported. Following cases of liver damage that even required transplantation, in November 2020, the EMA recommended limiting the prescription of UPA 5 mg (Esmya or generic medicines) as much as possible. So, currently, Esmya and generic medicines containing UPA 5 mg are only allowed to treat uterine fibroids in premenopausal women for whom surgical procedures (including uterine fibroid embolisation) are not appropriate or have not worked. On the other hand, these medicines must not be used for controlling the symptoms of uterine fibroids in the pre-surgical phase. Besides, it had already been demonstrated that in the women that had been pre-surgically treated with UPA, the myomas appeared softer and showed less clear cleavage planes. So, the result was that it was less easy to enucleate if compared to the enucleation modalities of the myomas belonging to women not pre-surgically treated with UPA [29]. In addition to all this, after the patients stop taking the UPA, leiomyomas revert [30,31]. Nowadays, hysterectomy remains the definitive treatment against uterine fibroids. In fact, at the moment, uterine leiomyomas represent the most common indication for hysterectomy in the world. However, it represents itself an additional problem concerning uterine fibroids and also the less invasive myomectomy leads to a serious postoperative morbidity [32,33]. Hysterectomy exerts also a very significant economic impact on the healthcare system all over the world, reaching an amount almost equal to $2.2 billion/year for the United States of America alone [34].

According to their anatomical location, uterine fibroids can be classified into three different types: submucosal fibroids, intramural fibroids and subserosal fibroids [35].

Uterine fibroids present themselves as solid, rounded masses, with an inhomogeneous eco-structure [36].

From a histological point of view, uterine fibroids can be classified into different types: usual leiomyoma, cellular leiomyoma that shows increased cellularity [37], lipoleiomyoma that exhibits adipocytes [38], apoplectic leiomyoma that shows stellate zones of recent hemorrhage [39] and rare, bizarre leiomyoma [40,41]. Among them, the usual leiomyoma is the most common histological variant with an incidence equal to approximately 94% and it is what is commonly referred to as a leiomyoma unless otherwise specified.

Usual leiomyomas are the ones considered a fibrotic disorder [42,43].

The leiomyomas were described as typical fibrotic tissues because they exhibit the upregulation of the extracellular matrix (ECM) proteins—above all, collagen 1A1, fibronectin and versican [44]. In particular, numerous authors showed that the uterine fibroids contain approximately 50% more ECM than the corresponding myometrium [45,46,47,48,49]. In addition, the ECM was suggested to represent a reservoir for growth factors, cytokines, chemokines, angiogenic and inflammatory response mediators, and proteases [43,50,51,52,53], which are all molecules thought to be involved in the initiation and development of the uterine fibroids [11].

In this regard, a very important matter about uterine fibroids is that their etiopathogenesis has not yet been clarified [53].

Nowadays, some major risk factors associated with the uterine leiomyomas are known and, among them, the following are the most important ones: early menarche, nulliparity, age (meaning late reproductive years), polycystic ovary syndrome, diabetes, hypertension, obesity, and heredity [10,50,54]. In addition to this, since black women have a high incidence rate of uterine leiomyomas [4], ethnicity may also be considered as a potential risk factor for this pathology.

Of the most important factors involved in the pathogenesis of uterine leiomyoma, in the literature, it has been reported that chromosomal abnormalities, both at the level of alterations of karyotypic character and at the level of alterations of cytogenetic character, are present in about 50% of leiomyomas [55,56,57]. In addition, in the leiomyomas, the chromosomes 2, 3, 6, 7, 8, 10, 11, 12, 13, 14 and 22 were demonstrated to present genetic alterations with the genes MED12, HMGA2, HMGA1, FH, BHD, TSC2, PCOLCE, ORC5L, and LHFPL3 supposed to be mutated in some way [50,58,59,60,61,62,63,64,65,66]. Mutations at the level of these genes and, in particular, MED12 mutation, FH inactivation and HMGA2 overexpression, as well as COL4A6-COL4A5 deletion were confirmed also by studies based on the modern high-throughput sequencing techniques [67].

Furthermore, as well as genetic factors, molecules and cellular events belonging to typical epigenetic pathways, such as several microRNAs, DNA methylation and histone modification, have also been described to be involved in leiomyomas [68,69,70,71]. In particular, uterine leiomyomas have been shown to present a dysregulation about a lot of different microRNAs and, among them also let7, miR-21, miR-93, miR-106b, and miR-200 and their predicted target genes. In addition, the same type of dysregulation has not been found in the healthy myometrium [68,72,73,74,75,76,77,78]. In addition, other potential gene-markers for the uterine leiomyoma can be provided through the use of gene set enrichment analysis [79].

Moreover, it has been clearly highlighted that estrogens and progesterone, the most important female hormones, as well as their correspondent receptors, exert a very relevant effect on uterine leiomyoma growth, and it was shown that, in doing this, the action of these molecules undergoes the mediation of other molecules such as growth factors, cytokines, and chemokines [11,80]. Sometimes, in the postmenopausal period, women need hormone replacement treatment (HRT) based on estrogens and progesterone in order to cope with some of the typical menopausal symptoms. So, also in postmenopausal women affected by uterine leiomyoma, estrogens and progesterone due to HRT can exert an important effect on uterine leiomyoma growth. For this reason, the use of these hormones should be limited [81].

Epidermal growth factor (EGF), heparin-binding epidermal growth factor (HB-EGF), platelet-derived growth factor (PDGF), insulin-like growth factor (IGF), transforming growth factor-alfa (TGF-α), transforming growth factor-beta (TGF-β), vascular endothelial growth factor (VEGF), acidic fibroblast growth factor (acidic-FGF), basic fibroblast growth factor (basic-FGF), activin and myostatin are the most important growth factors that mediate the estrogens and progesterone action within the uterine leiomyoma physiology [54,80,82,83,84]. In addition to this, interleukin (IL)-1, IL-6, IL-11, IL-13, IL-15, tumor necrosis factor-alfa (TNF-α), granulocyte macrophage-colony-stimulating factor (GM-CSF) and erythropoietin (EPO) are all cytokines that interact with estrogens and progesterone, playing an important role in uterine leiomyoma growth [85,86,87,88]. Additionally, chemokines, with their receptors and in particular, macrophage inflammatory protein (MIP)-1α, MIP-1β, regulated on activation normal T cell expression and presumably secreted (RANTES), Eotaxin, Eotaxin-2, IL-8, chemokine CC-motif receptor (CCR) 1, CCR3, CCR5, C-X-C chemokine receptor (CXCR) 1, CXCR2 and monocyte chemoattractant protein-1 (MCP-1) stimulate the uterine leiomyoma growth after the interaction with estrogens and progesterone [88,89].

**Figure 1:**
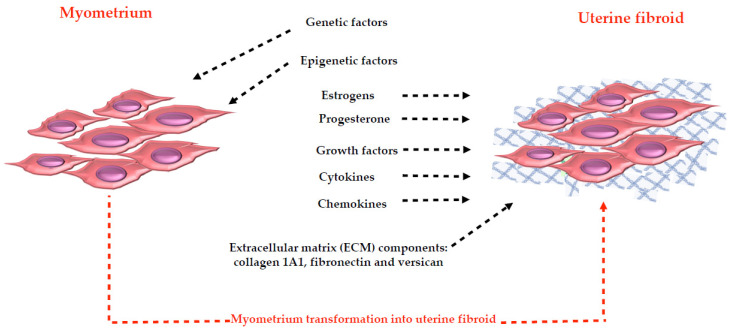
Illustration of the promoters of fibroid growth. The blue net represents the typical extracellular matrix (ECM) proteins: collagen 1A1, fibronectin and versican. The abundant ECM in uterine fibroids (approximately 50% more than the corresponding myometrium) was suggested to represent a reservoir for the other promoters of fibroid growth.

So, not only were growth factors [54,80,82], cytokines [11], chemokines [89], inflammatory response mediators [90], proteases [43,91,92,93] and the ECM, in particular as a reservoir of these molecules [43,50,51,52], shown to represent important actors in the establishment and in the growth of uterine fibroids [11], but also genetic alterations [50,55,64,94,95] and epigenetic mechanisms [69,70] as well as estrogens [96,97] and progesterone [97,98,99,100,101,102,103,104] can be considered as promoters of fibroid growth (Figure 1).

So far, we have discussed the anatomical environment and the histological features of the uterine fibroids as well as their incidence, their heavy symptoms, the available treatments and those still under study, the risk factors and also what is known about their pathogenesis. In this review, we will continue the discussion, thoroughly summarizing the role of the inflammatory process in uterine fibroid development and growth with particular regard towards the importance of the macrophages and the immune response in the uterine fibroids, trying to contribute to shed light on their etiopathogenesis.

The inflammatory process seems to have a noteworthy role in the establishment of the uterine fibroids [105]. In fact, we have just mentioned that the leiomyomas were described as typical fibrotic tissues [44] with a great deal of ECM [45,46,47,48].

In general, the fibrotic response arises from the recruitment of inflammatory cells such as monocytes and macrophages by means of inflammatory signals into the site of injury in every tissue and the consequent activation of fibroblasts that start producing collagen [106].

These fibroblasts are usually activated by inflammatory signals and they differentiate into myofibroblasts. They head the ECM turnover [107], leading to tissue homeostasis restoration [108,109]. A dysregulation in the myofibroblasts action can generate pathological fibrosis [106]. In fertile women, transient inflammation is a physiological and important process for the correct achievement of menstruation, ovulation, and parturition. An altered response can produce chronic inflammation in the uterus, ultimately leading to dysregulated tissue repair [90]. In particular, about leiomyoma development and growth, Leppert and her group suggested a model according to which, after a tissue injury, an abnormal response to tissue repair could occur, leading to disordered healing [42]. In a leiomyoma, smooth muscle cells, as well as fibroblasts or stem cells, can gain a myofibroblastic phenotype. In a dysregulated process, after myofibroblast transformation, the myofibroblasts cannot undergo apoptosis with the consequent formation of an altered ECM [30], which is a distinctive trait of the leiomyomas [44,45,46,47,48]. About this, it was noticed that fibroids exhibit a remarkable similarity to keloids, especially because of the disordered appearance of ECM and dysregulation of many genes in the ECM. In fact, microarray experiments have shown that fibroids possess gene features that resemble keloids [42]. So, fibroids could represent a disorder of wound healing and could arise in response to dysregulated extracellular signals as well as keloids [42]. Additionally, myomectomy and caesarean section, which have already been demonstrated to be causes of uterine rupture, may themselves represent a kind of damage followed by a wound healing response. In women showing disordered extracellular signals because of these alterations, a fibroid may develop [110].

## 2. The Role of Macrophages in Tissue Repair and Fibrosis in Several Organs

**Figure 2:**
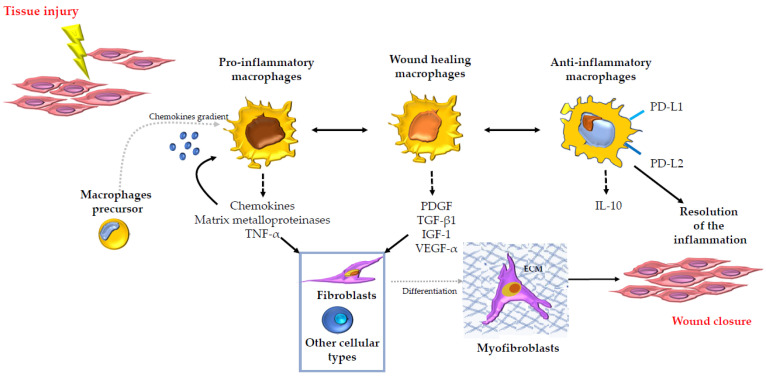
Illustration of the role of the macrophages and their highly flexible programming in tissue repair and fibrosis in several organs. Macrophages, because of their high flexibility, can play a key regulatory role in every stage that characterizes the tissue repair and fibrosis from the promotion to the resolution of the inflammation leading to the wound closure. The figure shows the principal events and principal molecules: chemokines, Matrix metalloproteinases, tumor necrosis factor-alfa (TNF-α), platelet-derived growth factor (PDGF), transforming growth factor-beta 1 (TGF-β1), insulin-like growth factor-1 (IGF-1), vascular endothelial growth factor-alfa (VEGF-α), programmed death-ligand 1 (PD-L1) and programmed death-ligand 2 (PD-L2), interleukin-10 (IL-10) involved in the process, highlighting the different phenotypic states that the macrophages can assume in the process. The blue net represents the extracellular matrix (ECM) that is produced by myofibroblasts after that fibroblasts or other cellular types differentiated into them.

Although a lot of different cellular types such as fibroblasts, epithelial cells, endothelial cells, stem cells, neutrophils, innate lymphoid cells (ILCs), NK cells, B cells and T cells join to the complex inflammatory response that leads to tissue repair [109], macrophages develop a key regulatory role in every stage that characterizes the tissue repair and fibrosis [111]. This capability could be due to the macrophages’ highly flexible programming [112]. In fact, within the injured tissue, the macrophages can be found in several different phenotypic states, and this flexibility allows them to perform many functions beginning from the promotion and resolution of inflammation, including the removal of apoptotic cells, up to the support of cell proliferation following injury [113]. After tissue injury, through chemokine gradients and some different adhesion molecules, a lot of inflammatory monocytes and macrophage precursors are recalled from the bone marrow to the injured site. These recruited cells outnumber the resident tissue macrophages [114,115]. At this point, the release in the local tissue microenvironment of cytokines and growth factors represents the signal for the proliferation of both the recruited and resident macrophages [116,117]. In addition, in response to these signals, the macrophages also change their aspect in order to develop their functions [116,117]. In this way, macrophages assume the phenotype that could be called “pro-inflammatory macrophages” and so they can lead the initial phase of the response to injury since they represent an important source of chemokines, matrix metalloproteinases and other inflammatory mediators such as TNF-α [111]. The inflammatory process in response to injury goes on because of the macrophages’ high flexibility [112]. In fact, they assume the phenotype that could be called "wound healing macrophages", which are specialized in the production and consequent secretion of several growth factors such as PDGF, transforming growth factor-beta 1 (TGF-β1), insulin-like growth factor-1 (IGF-1) and vascular endothelial growth factor-alfa (VEGF-α) [118,119,120,121,122]. These molecules stimulate cell proliferation and angiogenesis [118,119,120,121,122]. In addition, under the effect of the soluble mediators produced by the wound healing macrophages, local and recruited tissue fibroblasts are induced to differentiate into myofibroblasts that drive the wound contraction and closure especially through the synthesis of extracellular matrix components [123] such as collagen 1A1, fibronectin and versican. Wound healing macrophages develop their regulatory role [111] also towards neighboring parenchymal and stromal cells’ proliferation and expansion, and they can recruit additional stem cell and local progenitor cell populations in order to make them join to tissue repair in case of severe injury. At this point, the macrophages again change their aspect, gaining another phenotype, which can be called "anti-inflammatory macrophages" [124]. Anti-inflammatory macrophages act in response to several inhibitory mediators such as IL-10 and in turn they release a wide range of anti-inflammatory mediators such as IL-10 and TGF-β1 and show as cell surface receptors the proteins programmed death-ligand 1 (PD-L1) and programmed death-ligand 2 (PD-L2), which represent the principal molecules involved in the immune system suppression and in the resolution of the inflammation [125,126,127,128] (Figure 2). Therefore, wound healing is a process that must be tightly regulated, otherwise it may lead to the formation of chronic wounds that in turn may facilitate the development of pathological fibrosis [129]. The macrophages, with their great flexibility that allows them to adopt different phenotypes [112,113], could play a unique, important and critical role at each stage of the wound healing, from the initiation and maintenance up to the resolution of the tissue repair process. Different studies have highlighted the macrophages’ great flexibility. In the literature, this plasticity is often reported as the M1/M2 dichotomy of macrophages. It describes the different macrophage subtypes that are involved in the tissue repair process. The M1/M2 dichotomy describes the macrophage subsets showing the M1 subtypes expressing higher levels of several pro-inflammatory cytokines, such as TNF-α and interleukin-1 beta (IL-1β) and the M2 subtypes expressing increased levels of anti-inflammatory cytokines, such as IL-10 and TGF-β [130,131,132]. Even if this is a widespread nomenclature, it is now thought that the M1/M2 dichotomy is not sufficient at all to describe the several different phenotypes and functions of macrophages in vivo [133], also because both M1 and M2 markers can often be expressed at the same time [134]. In addition to this, studies about tissue repair in skeletal muscle showed that in vivo macrophage activation signaling pathways do not correspond to in vitro M1/M2 ones. Among them, for example, we can mention the signal transducer and activator of transcription 1 (STAT1)/interferon gamma (IFN-γ) receptor [135], canonical M2 markers induced by IL-4 [135], the transducer and activator of transcription 6 (STAT6) in IL-4 signaling [136], the IL-4/IL-13 signaling [137], and last but not least, hypoxia-inducible factors (HIFs) in M1/M2 gene expression [138] and in macrophage accumulation [139] pathways. Therefore, it can be affirmed the M1/M2 macrophage dichotomy was conceived by studying macrophages in culture and it is not suitable in order to describe macrophages in vivo straightforward [140]. The most noteworthy concept we have to focus on is that both definitions of M1/M2 macrophages and pro-inflammatory/wound healing/anti-inflammatory macrophages agree with the fact that macrophages have a great flexibility so that they can assume several different phenotypes [112,113], and this capability may enable them to lead the tissue repair process. Indeed, something dysregulated such as macrophage proliferation, accumulation and infiltration, within the reported macrophage action could lead to uncontrolled repair tissue and to the consequent pathological fibrosis. Several studies have been carried out in order to characterize the macrophages’ behavior within the initiation, maintenance and resolution of the tightly regulated wound healing response in different organs.

## 3. Macrophages in Uterine Fibroids

As it has been mentioned before, inflammation plays an important role in the pathophysiology of the uterine leiomyoma [105], which was defined as a typical fibrotic tissue [42,43].

Several studies have highlighted the involvement and importance of the macrophages in the inflammation and consequent fibrosis that are typical features of leiomyoma tissue [42,43,44,45,46,47,48,89,90,105,106].

Through the use of the glycosylated transmembrane glycoprotein antigen (CD68) that belongs to a family of lysosomal granules [141] as a marker of mature and activated macrophages, Miura et al. studied the macrophages’ infiltration in different types of uterine leiomyomas. They demonstrated the myoma nodules and the autologous endometrium of the submucosal myomas (SMM) and intramural myomas (IMM) show a higher level of macrophage infiltration compared to the corresponding tissues of the subserosal myomas (SSM) or to the eutopic endometrium belonging to women without uterine myomas used as a control [142]. In addition to this, the authors showed a similar pattern also for the MCP-1 concentration. Moreover, MCP-1 concentration was shown to be positively correlated with the macrophage infiltration in SMM and IMM myoma nodules and endometrium [142]. So, the overproduction of MCP-1, which is one of the most important chemokines involved in the monocytes’/macrophages’ migration and infiltration [143], may represent the cause of the macrophages’ infiltration in women with SMM and IMM, and this accumulation of inflammatory macrophages could lead to a negative effect on reproductive outcomes in women with SMM or IMM [142]. Anyway, the increased infiltration and accumulation of macrophages within some subtypes of fibroid tissue may represent proof of the macrophages’ importance within leiomyoma pathology.

In support of all this, Khan and colleagues demonstrated that endometria belonging to women with uterine fibroids undergoing gonadotrophin-releasing hormone agonist (GnRHa) therapy exhibited decreased values of macrophage infiltration and MCP-1 levels when compared to corresponding values of macrophage infiltration and MCP-1 levels in endometria belonging to women with uterine fibroids that had not undergone GnRHa therapy [144].

On the other hand, a previous study conducted by Sozen highlighted that the myometrium of the women with uterine fibroids taking GnRHa and in particular the endothelial cells of blood vessels in myometrial tissues surrounding the leiomyoma show higher MCP-1 levels than the myometrium of the women with uterine fibroids not taking GnRHa [145]. This difference was not accompanied by a significant difference in the number of tissue macrophages between women who had undergone GnRHa therapy and women who had not undergone GnRHa therapy [145]. In this study, Sozen and colleagues expected to detect a macrophage infiltration increase following the MCP-1 increase because of the GnRHa use, but these results were disproved [145]. It is known that the uterus after GnRHa exposition shows a reduced arterial blood flow [146,147] and this may impair the macrophage accumulation, representing the explanation for why a macrophage infiltration increase does not accompany the MCP-1 increase in the myometrium of the women with uterine fibroids taking GnRHa [145].

In addition, estrogens and progesterone, which are recognized to be important promoters of the leiomyoma growth [96,97,98,99,100,101,102,103,104], impair MCP-1 expression [89].

Therefore, the discrepancy between the results obtained by Khan [144] and the results obtained by Sozen [145] may be due to the use of different tissue types or to the difference in tissue specificity and number of analyzed samples. The most important point to focus on is that within the complex network of molecules that are involved in the leiomyomas’ development and growth, MCP-1 can also carry out an important role, taking part in the regulation of the macrophage infiltration. The MCP-1 regulatory action on macrophage infiltration may in turn be important for the development of the uterine fibroids. In addition to this, the cited studies about GnRHa, which is known to be commonly used for the treatment of uterine myomas, testify in any case that macrophages represent an aspect to be taken into consideration for the treatment and further clarification of the etiopathogenesis of uterine fibroids.

In addition, Kitaya and Yasuo have provided further evidence of the involvement and importance of the macrophages in the pathology of uterine fibroids. They analyzed the leukocyte density and composition in the human cycling endometrium in women affected by uterine fibroids. By immunohistochemical analysis, the authors compared endometrium with neighboring nodules with autologous endometrium without neighboring nodules and with allogeneic endometrium belonging to women without uterine fibroids. In particular, the macrophage (CD68 positive cells) density is significantly higher in the endometrium close to the leiomyoma nodules compared to the autologous endometrium far from the leiomyoma, as well as compared to the allogenic endometrium of women without uterine fibroids in the mid-to-late secretory phase [148]. The authors reported also that the endometrium far from the leiomyoma nodules had more macrophages than the endometrium of women without uterine fibroids in the proliferative and late secretory phase [148].

In addition, according to the results obtained by Miura et al. [142], the authors highlighted that the macrophage density is significantly higher in SMM than in IMM and SSM [148].

In addition, Kitaya and Yasuo showed that the whole stromal pan-leukocyte density is altered in the endometrium containing neighboring nodules of the women affected by uterine fibroids. Above all, they highlighted that the increased stromal pan-leukocyte density in endometrium with neighboring nodules during the proliferative phase is largely due to the increased macrophage density [148]. These findings testify once again that macrophages represent a very important aspect within the pathology of uterine fibroids.

Another aspect that is important to highlight about the involvement of the macrophages in uterine fibroids is the GM-CSF expression in leiomyoma and in myometrium. In fact, this cytokine represents the most important growth factor for macrophage proliferation, differentiation and functional activation [149].

In addition to this, GM-CSF has been demonstrated to determine the fibrotic reaction in several tissues [150,151,152,153,154]. In particular, thinking about the association between the overexpression of TGF-β and the establishment of tissue fibrosis through the stimulation of the conversion of fibroblasts into myofibroblasts in various sites throughout the body [155,156,157], GM-CSF has been shown to be involved in a fibrotic process that includes the accumulation of α smooth muscle actin-rich myofibroblasts through a mechanism involving TGF-β expression [150,151,152,153,154,157]. In addition, bearing in mind that it was demonstrated that TGF-β synthesis and release are increased in uterine fibroids [158], all of this makes GM-CSF one of the most important cytokines that may be able to play a key role in the initiation and maintenance of uterine leiomyoma, which is a typical fibrotic disorder [44].

In addition, since GM-CSF is considered to be the most important growth factor for macrophage proliferation, differentiation and functional activation [149], we could think that GM-CSF action and macrophage infiltration as they have been previously described could be interconnected within the development of the uterine fibroid pathology.

Considering the relationship between macrophages and uterine fibroids development, it is relevant that TGF-β is involved in tissue fibrosis in several sites throughout the body [155,156], is overexpressed in leiomyomas [158], and at the same time is the most important growth factor produced by macrophages [159]. In addition, TGF-β contributes to myofibroblast transformation [159], which represents another important aspect leading to the development of uterine fibroids [42,53].

**Figure 3:**
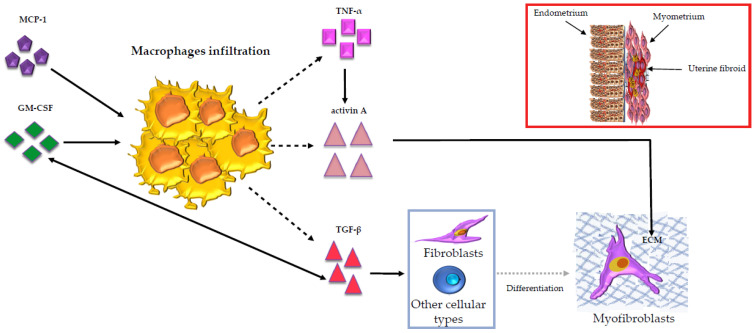
Illustration of the macrophages’ (yellow in the figure) role in uterine fibroids. Monocyte chemoattractant protein-1 (MCP-1) takes part in the regulation of the macrophages’ infiltration. The granulocyte macrophage-colony-stimulating factor (GM-CSF) is considered the most important growth factor for macrophage proliferation. GM-CSF can establish regulatory interactions with the transforming growth factor-beta (TGF-β), which was shown to be the most important growth factor secreted by macrophages. In uterine fibroids, TGF-β is overexpressed and it contributes to myofibroblast differentiation. Macrophages also secrete activin A, an immuno-regulator belonging to the TGF-β family. Activin A develops a pro-fibrotic action leading to the expression of the extracellular matrix (ECM) proteins (represented by the blue net in the figure), which are overexpressed in uterine fibroids. Activin A mRNA expression in uterine fibroids is upregulated by the tumor necrosis factor-alfa (TNF-α), an inflammatory mediator mainly produced by macrophages. On the right, above the image that portrays the myofibroblasts, the uterine fibroids (red in the figure) with the myometrium (pink in the figure), the endometrium (brown in the figure), the macrophages (yellow in the figure) and the overexpressed ECM proteins (blue net in the figure) are represented. The blood vessels within endometrium are also represented (red lines in the figure).

It is very important to highlight that macrophages secrete not only TGF-β, which plays a key role in the progression of the fibrosis [159], but also produce activin A, an immunoregulator belonging to the TGF-β family [160]. Our group showed that, in primary leiomyoma cells, activin A acts as a pro-fibrotic factor leading to the expression of ECM proteins [161] that are upregulated in leiomyoma [44]. In addition to this, we later demonstrated in leiomyoma that activin A mRNA expression is upregulated by TNF-α [53] according to the literature, where the same effect is reported also in human bone marrow stromal cells and monocytes, human bone marrow stromal cell lines, cultured fibroblasts and keratinocytes [162,163,164,165]. The most remarkable aspect about activin A upregulation by TNF-α is that TNF-α is also mainly produced by macrophages [166] (Figure 3).

**Figure 4:**
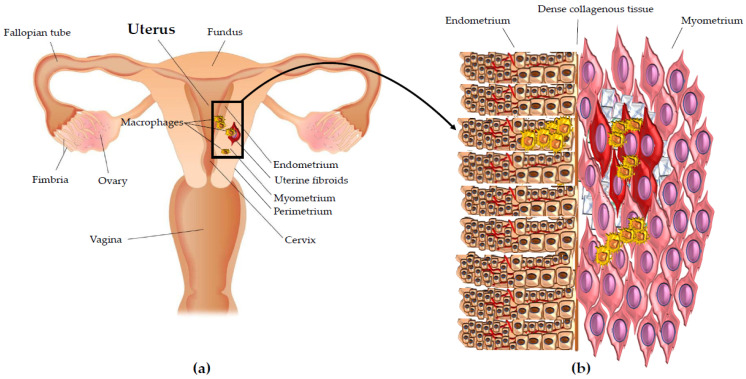
Macrophages in uterine fibroids. (**a**) Illustration of uterus showing the macrophage density in uterine fibroids pathology. (**b**) Enlargement of the detail showing the macrophage density in uterine fibroids pathology. Macrophages (yellow in the figure) predominantly localize inside uterine fibroids (red in the figure) and in the myometrium tissue (pink in the figure) next to them. Autologous distant myometrium shows low levels of macrophage infiltration. The macrophage density is higher also in the endometrium (brown in the figure) next to uterine fibroids than in the autologous endometrium far from uterine fibroid nodules. The extracellular matrix (ECM) around and within the uterine fibroids is also represented (blue net in the figure). The blood vessels within endometrium are also represented (red lines in the figure).

Studying leiomyomas, we found, according to the other results reported in this paper, that macrophage infiltration inside the leiomyoma is significantly higher compared with autologous myometrium more than 1.5 cm from the leiomyoma [53]. More precisely, by CD68 staining, our group found that macrophages predominantly localize inside leiomyoma and in the myometrium tissue next to leiomyoma. On the contrary, autologous distant myometrium showed low levels of CD68-positive macrophages [53] (Figure 4a,b). So, these findings highlight unequivocally the importance of inflammation, and above all, the key role of the macrophages in the development and growth of uterine fibroids.

Since these results were obtained by studying different histotypes of leiomyomas, we could add that the reported macrophage localization is valid for both cellular and usual leiomyomas with the cellular leiomyoma showing higher levels of CD68-positive macrophages compared with usual leiomyoma [53].

**Figure 5:**
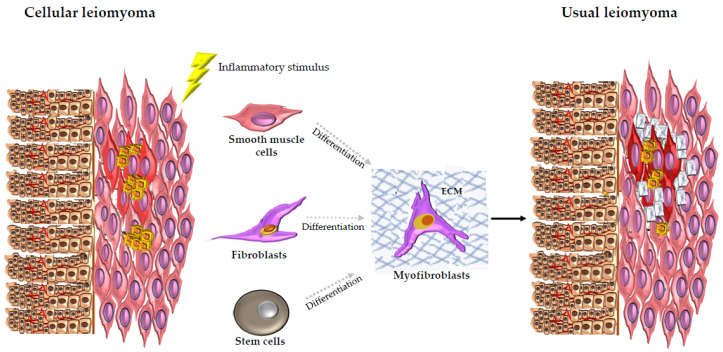
Illustration of the possible phase mechanism of leiomyoma development proposed by our group. Cellular leiomyoma is considered as the first step in the tumoral transformation. In fact, cellular leiomyoma shows higher levels of macrophage (yellow in the figure) infiltration and an increased number of inflammatory cells. This aspect could represent a response to an inflammatory stimulus that leads some cellular leiomyoma cells to myofibroblast differentiation with the consequent upregulation of the typical extracellular matrix (ECM) proteins. In fact, usual leiomyoma shows a larger amount of ECM proteins and low levels of macrophage infiltration. So, usual leiomyoma could be considered as the late-phase tumor. The blue net represents the typical ECM proteins: collagen 1A1, fibronectin and versican. The red color represents the uterine fibroids (light red for cellular leiomyoma histotype and dark red for usual leiomyoma histotype). The pink color represents the myometrium; the brown color represents the endometrium. The blood vessels within endometrium are also represented (red lines in the figure).

So, our group proposed a possible phase mechanism for the leiomyoma development. According to this mechanism, cellular leiomyoma histotype, as also suggested by Dixon et al. [54], could be considered as the first step in the tumoral transformation. In fact, cellular leiomyomas show low levels of the typical ECM proteins. On the other hand, our group noticed that cellular leiomyomas, in addition to higher levels of CD68 positive macrophages, also have an increased number of leukocytes and mast cells that are other types of inflammatory cells [53]. This aspect could represent a response to an inflammatory stimulus. As a result, these first step cells of the cellular leiomyoma undergo myofibroblast differentiation with the consequent upregulation of the typical ECM proteins [53]. In fact, usual leiomyomas that could be considered as the late-phase tumor show a larger amount of ECM proteins than we observed [53] (Figure 5).

So, the data published by our group provide additional proof of the involvement of inflammation and the importance of the macrophages’ action in the pathophysiology of uterine fibroids.

## 4. Conclusions

About uterine fibroids, whose etiopathogenesis has not yet understood at all, a deregulated inflammatory process leading to an exaggerated tissue repair may explain the abundant ECM, a typical feature of the uterine fibroids that, just because of this characteristic, is considered a typical fibrotic tissue. In particular, a key role in this complex network can be played by the macrophages when a deregulation in their action happens. In fact, the macrophages are important tissue repair actors by means of their highly flexible programming and their consequent plasticity. So, during the inflammation process and the consequent wound healing that the inflammatory mechanisms lead to, macrophages have to proliferate and infiltrate within the damaged tissue; then, they assume different phenotypes and produce molecules that start, drive and finally stop the tissue repair up to the wound closure. So, there are a lot of critical checkpoints that need to be tightly regulated. In the uterine fibroids, sufficient proof of deregulated macrophage action was provided. In fact, increased infiltration and accumulation of macrophages within some subtypes of fibroid tissue were demonstrated. In addition to this, the importance of cytokines and chemokines such as GM-CSF and MCP-1 for the proliferation and infiltration of the macrophages in the uterine fibroids was shown. Furthermore, their expression in leiomyomas has been altered. All this, in turn, has an impact on the molecules that are secreted by macrophages. Among these molecules, the inflammation mediator TNF-α and the growth factors activin A and TGF-β can be considered the most important ones because they are known to be involved in the fibrosis that characterizes the uterine fibroids. In addition, these molecules, secreted by macrophages, were demonstrated to be interconnected with each other and with the GM-CSF. In this way, they establish in the uterine fibroids a complex network that, because of a dysregulation, at one or more levels, may explain the mechanisms that occur from an excessive wound healing driven by the inflammatory process to the fibrosis.

Better understanding the process leading to the increased infiltration and accumulation of macrophages in leiomyomas and the molecules involved within the consequent exaggerated tissue repair that arises from it, may represent proof of the macrophages’ importance for the leiomyoma pathology. All this can also contribute to shed light on uterine fibroids etiology.

In turn, better understanding the uterine fibroids’ etiology may represent the starting point to identify possible new therapy targets. This could improve the quality of life of the women affected by uterine fibroids.

Last but not least, a therapy against this pathology could also bring about better outcomes for pregnant women affected by this pathology.

## Data Availability

Not applicable.
